# Myopathy due to a creatine deficiency disorder in a family of mixed breed dogs with a glycine amidinotransferase gene mutation

**DOI:** 10.1093/jvimsj/aalaf055

**Published:** 2026-01-21

**Authors:** Hugo Leonardi, Katie M Minor, Julien Fritz, Steven G Friedenberg, Jonah N Cullen, Ling T Guo, G Diane Shelton

**Affiliations:** Azurvet Veterinary Center, Saint-Laurent-du-Var, France; Department of Veterinary Clinical Sciences, College of Veterinary Medicine, University of Minnesota, St Paul, MN 55108, United States; Azurvet Veterinary Center, Saint-Laurent-du-Var, France; Department of Veterinary Clinical Sciences, College of Veterinary Medicine, University of Minnesota, St Paul, MN 55108, United States; Department of Veterinary Clinical Sciences, College of Veterinary Medicine, University of Minnesota, St Paul, MN 55108, United States; Comparative Neuromuscular Laboratory, Department of Pathology, School of Medicine, University of California San Diego, San Diego, CA, United States; Comparative Neuromuscular Laboratory, Department of Pathology, School of Medicine, University of California San Diego, San Diego, CA, United States

**Keywords:** creatine deficiency disorder (CDD), arginine:glycine amidinotransferase (AGAT), glycine amidinotransferase (*GATM*), canine inherited myopathy, megaesophagus

## Abstract

**Background:**

Myopathies caused by genetic abnormalities are increasingly recognized in veterinary medicine.

**Hypothesis/Objectives:**

Clinically and genetically characterize a novel creatine deficiency disorder (CDD) myopathy in a family of mixed breed dogs.

**Animals:**

Three siblings from the same litter were evaluated and genetically tested, including 2 dogs that were clinically affected and one dog clinically normal. All dogs were client owned.

**Methods:**

Case series describing clinical, imaging, electrodiagnostic, histopathologic investigations, and response to treatment. Whole genome sequencing and bioinformatics were performed to identify a causative variant followed by Sanger sequencing to confirm the suspected variant in related dogs.

**Results:**

Clinical signs included megaesophagus with generalized muscle atrophy in both affected dogs. One dog showed exercise intolerance. Computed tomography (CT) scan revealed bilateral and symmetrical diffuse hypoattenuating muscle lesions. Electromyography was characterized by nonspecific abnormal spontaneous activity in electrodiagnostically affected muscles. Type 2 fiber atrophy and excessive intramyofiber lipid droplets in type 1 muscle fibers were the predominant findings in histopathology. Both affected dogs were homozygous for a unique *GATM* p.R414C (NP_001274013.1) missense variant, while the unaffected sibling did not have this variant. All clinical signs improved after 3 days of creatine (800-1500 mg/kg/day) and L-carnitine (80-150 mg/kg) supplementation and remained stable at the time of writing 4 months after diagnosis.

**Conclusions and clinical importance:**

This is a report of CDD in dogs characterized by a glycine amidinotransferase (*GATM*) variant, which showed a good short-term outcome with supplementation with creatine and L-carnitine.

## Introduction

Genetic myopathies are a heterogenous group of inherited disorders that affect the skeletal muscles, leading to progressive weakness, poor quality of life, and in some cases premature death. These conditions are increasingly recognized in veterinary medicine, particularly in dogs, where they can pose therapeutic challenges. Most cases have a poor long-term prognosis with limited treatment options to improve quality of life and lifespan.^1^

The concept of one health medicine and the translational approach from human medicine to the veterinary community have helped develop diagnostic and therapeutic tools for animals.^2^ Notably, advances in genetic testing in dogs allow for accurate identification and classification of inherited disorders and permits a comparative approach to known human inherited disorders.^3–5^ The aim of this study is to clinically and genetically characterize a novel creatine deficiency disorder (CDD) myopathy in a family of mixed breed dogs for which a comparative approach to a known human disorder was made for diagnosis and treatment options.

## Material and methods

### Animals

Two affected female littermates (dogs 1 and 2) were evaluated separately at approximately 2 years of age. Neurological examination of both affected dogs was performed by an ACVIM diplomate (Neurology) before initiating treatment. Four months after initiating treatments, a recheck examination and chest radiographs were performed on dog 1 and a phone reevaluation was done on dog 2. One unaffected male littermate (dog 3), with no apparent clinical signs, was evaluated by a general veterinarian. All dogs were client-owned. No information on the parents was available.

The complete medical record, including previous complete blood count (CBC), blood biochemistry, and 3-view chest radiographs, was available for the 2 affected dogs (dogs 1 and 2). Dog 1 underwent further investigations that included additional 3-view chest radiographs, lumbar spine to pelvic limb computed tomography (CT) scan, electrodiagnostic evaluation including electromyography (EMG) and motor nerve conduction study, serological blood testing for *Erlichia canis, Anaplasma phagocytophilum, Borrelia burgdorferi*, (SNAP 4 DX, IDEXX laboratory, ELISA), *Leishmania* sp. (SNAP LEISH, IDEXX laboratory), *Neospora* sp. (IgG and IgM, indirect immunofluorescence assay, AnyDiag laboratory), antigenemia testing for *Dirofilaria immitis* (SNAP 4 DX, IDEXX laboratory, ELISA), and collection of muscle biopsies. Ethylenediaminetetraacetic acid blood samples were collected for genetic testing in all 3 dogs. Both oral and written consent were obtained from dog owners to pursue diagnostic testing.

### Imaging

Dogs 1 and 2 underwent thoracic radiographs to evaluate megaesophagus at the time of diagnosis and 4 months after treatment for dog 1. Dog 1 underwent lumbar spine, pelvis, and stifle CT scan (64 slice Toshiba Aquilion 64, American Medical System, USA). Images were obtained before and after intravenous injection of Iopamidol 300 mg/mL.

### Electrodiagnostic testing

Dog 1 was anesthetized for electrodiagnostic testing that included EMG and motor nerve stimulation with calculation of the mean motor nerve velocity (Nicolet Viking Quest EMG recorder) as described.^6^ Electromyography was performed on the appendicular muscles of 1 thoracic and 1 pelvic limb, paraspinal muscles from the lumbar, thoracic and cervical spine, external laryngeal muscles, temporalis, and masseter muscles. A scale from 0 to 4+ was used to describe the severity of EMG finding depending on the density of the fibrillations or positive sharp waves noted per area tested in a single muscle, as previously described.^7^ Insulated monopolar stainless steel needle electrodes were used for nerve stimulation, muscle recording and ground.

### Histopathology

Dog 1 was anesthetized and muscle biopsy samples collected from multiple muscles by an open biopsy procedure. Muscle sampling was guided by abnormal electrodiagnostic findings and by muscles appearing abnormal on CT imaging. Unfixed chilled and formalin fixed biopsy samples were submitted to the Comparative Neuromuscular Laboratory, Department of Pathology, University of California San Diego, La Jolla, CA, USA, from the triceps, infrascapular, paravertebral lumbar, cranial tibial, and semitendinosus muscles. The unfixed biopsies were evaluated in frozen sections using a standard panel of histochemical stains and reactions^8^ and the fixed biopsies evaluated in routine paraffin sections. In addition to the routine stains and reactions, the combined reactions for cytochrome C oxidase (COX) and succinate dehydrogenase (SDH) were performed as a specific indicator of mitochondrial deletions.^9^

### Genetic testing

Genomic DNA was prepared from archived frozen muscle from dog 1 using the Qiagen DNEasy kit according to package instructions. DNA libraries were prepared using an Illumina TruSeq PCR-Free kit and 150 bp paired-end reads were generated on an Illumina NovaSeq 6000 sequencer by Azenta Life Sciences (South Plainfield, NJ 07080, USA). A total of 225 million paired-end reads were generated, corresponding to a mean 14-fold genome-wide coverage. Sequence reads were mapped against the dog reference genome UU Cfam GSD 1.0^10^^,^^11^ and processed using the OnlyWAG pipeline as described.^12^ Raw sequence reads are available in NCBI’s Short Read Archive at SRR33639174 BioProject PRJNA937381.

Whole genome sequencing (WGS) variants from the affected dog were compared to an internal WGS database developed at the University of Minnesota, St. Paul, MN, USA containing 3023 dogs, wolves, and coyotes of 402 diverse breeds; this database includes 1971 dogs, wolves, and coyotes released by the Dog10K consortium.^11^ The WGS data from these 3023 dogs were processed using the same bioinformatics pipeline referenced above. Variants unique to the affected dog were prioritized by predicted consequence and impact by variant effect predictor.^13^ High (eg, frameshift, loss or gain of stop or start codon, affecting a splice junction) and moderate impact (eg, missense) variants were evaluated for further investigation. A list of these unique variants is provided in Table S1.

### Sanger sequencing

Genotyping was performed via Sanger sequencing of a 380 bp PCR amplicon for the identified variant to confirm the suspected variant in related dogs. This PCR utilized standard conditions with forward primer 5′-ACCAGGCTTAAGACCCCTGT-3′ and reverse primer 5′-AAGACTGTTTGGGGTTCAGG-3′.

## Results

### Clinical/neurological findings

Since acquisition, the primary clinical signs reported by the owners included difficulty walking and chronic regurgitation in both dogs 1 and 2. Dog 1 had signs of exercise intolerance characterized by the inability to walk for longer than 20 minutes before collapsing, a bunny hopping gait while running and reluctance to walk up the stairs. Rare episodes of regurgitation were also reported that resolved after the owners split meals into smaller portions. Hiccups and pharyngeal sounds were occasionally noted after a meal. Dog 2 presented for cachexia and chronic daily episodes of regurgitation since acquisition without obvious paresis or exercise intolerance. Episodes of aspiration pneumonia were treated with courses of unknown antibiotics. In both dogs, neurological examination was normal except for a bunny hopping gait while running in dog 1. Physical examination revealed generalized amyotrophy that was mild in dog 1 and severe with cachexia in dog 2. A video of the gait and parts of the neurological examination of dog 1 is presented in Video S1 (Supplementary Data).

Laboratory evaluations including CBC, serum biochemistry analysis including electrolytes performed by the general practitioner did not reveal abnormalities 1 year (dog 2) and 1 week (dog 1) before presentation. Serum creatine kinase (CK) activity was normal in both dogs at the time of diagnosis.

Previous 3-view thoracic radiographs performed by the general practitioners revealed a megaesophagus in both dogs.

Serological testing was performed at the time of initial diagnosis on dog 1 and were negative for *E. canis, A. phagocytophilum, B. burgdorferi, Leishmania* sp. and *Neospora* sp. Antigenemia testing for *Dirofilaria immitis* was also negative.

### Imaging

**Figure 1:**
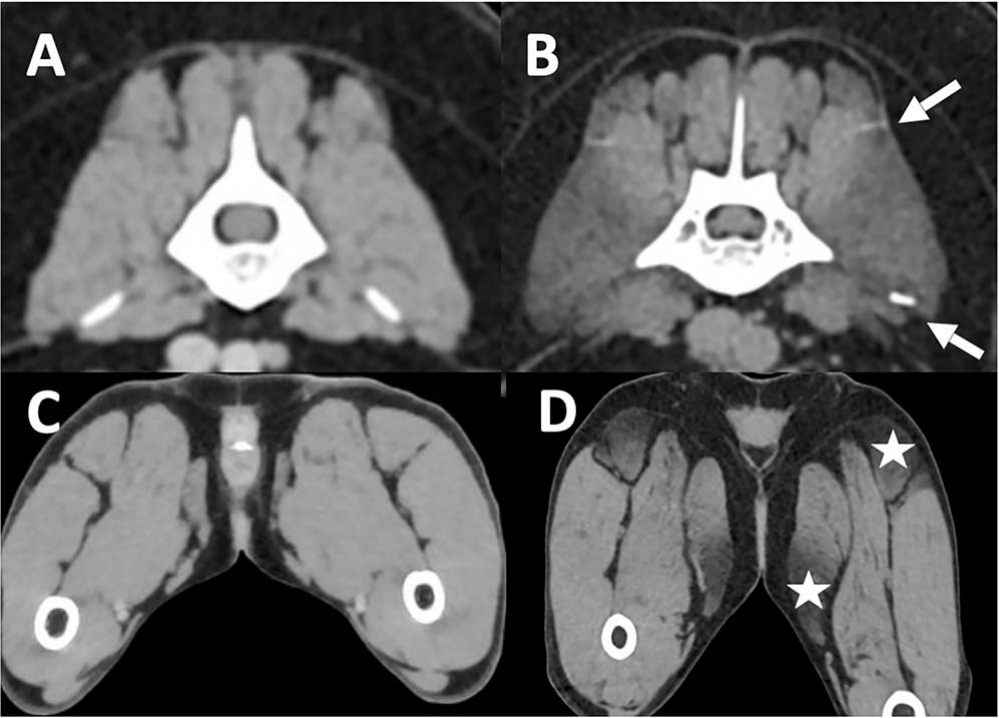
Soft tissue post-contrast CT scan transverse sections at the level of the lumbar vertebral column (A and B) and at the level of the pelvic limbs (C and D) of an affected dog (B and D) and a matched control (A and C). White arrows delineate the iliocostalis muscle that appears hypoattenuated on CT scan (B). White stars reveal hypoattenuating lesions in the semitendinosus and gracilis muscles (D).

Computing tomography scan of the lumbar spine and pelvic limbs in dog 1 revealed a generalized bilateral and symmetrical negative attenuation of the muscles, most notably on the iliocostalis lumborum, semitendinosus, and gracilis muscles with no contrast enhancement (Figure 1). The Hounsfield Unit (HU) of the muscle lesions was compatible with fatty tissue (40 to −70 HU).^14^

### Electrodiagnostic testing

**Figure 2:**
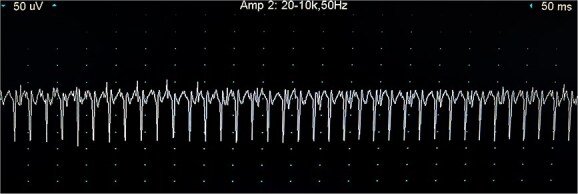
Train of positive sharp waves recorded during EMG measurement of the palmar interosseous muscle of dog 1. Each vertical unit represents 50 μV, each horizontal unit represents 50 ms. Abbreviation: EMG = electromyography.

**Figure 3:**
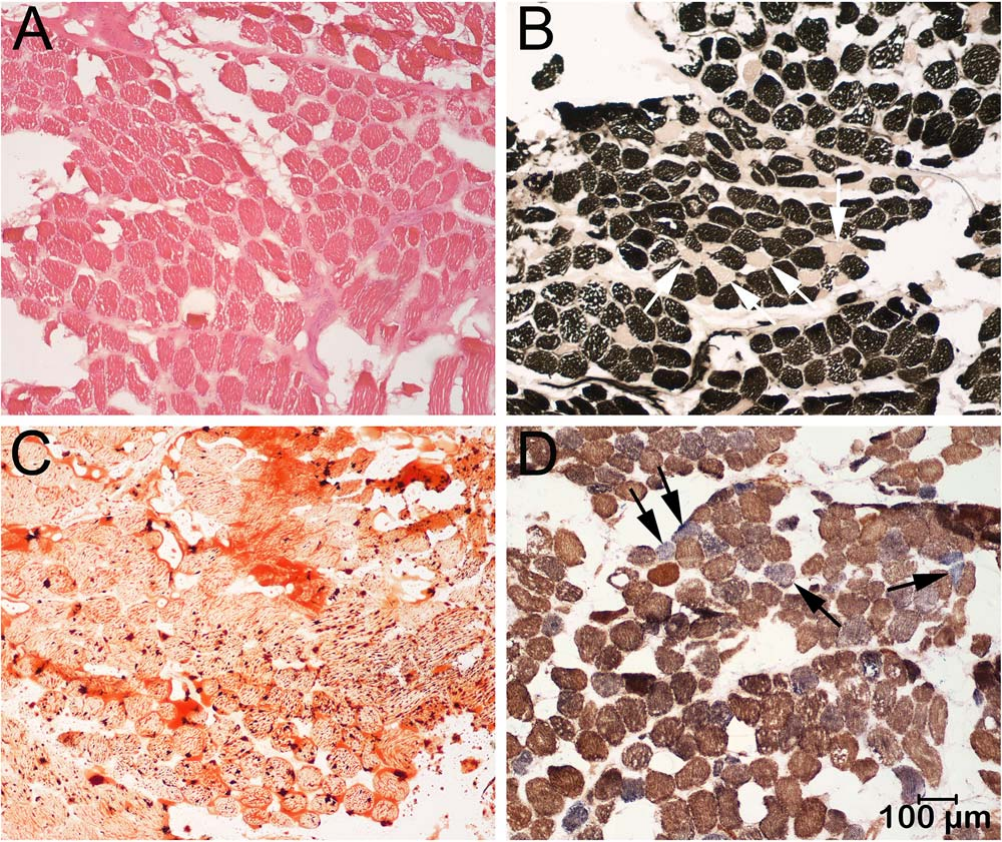
Frozen sections from the paravertebral muscle were evaluated by a standard panel of histochemical stains and reactions including the H&E stain for general morphology (A), the myofibrillar adenosine triphosphatase reaction for fiber typing (B, ATPase reaction at pH 4.3), the Oil red O stain for neutral triglycerides (C), and the combined cytochrome C oxidase and succinic dehydrogenase reaction as a specific mitochondrial reaction (D, COX/SDH). Excessive variability in myofiber size was present (A) with a type 1 fiber predominance (dark staining fibers), type 2 fiber atrophy (light staining fibers) and numerous clear vacuoles in type 1 fibers. Numerous and large intramyofiber lipid droplets were identified with the Oil red O stain (C). Sporadic muscle fibers (arrows) were blue stained with the COX/SDH reaction indicating loss of COX activity. Bar in *D* = 100 μm for all images. Abbreviations: COX = cytochrome C oxidase; H&E = hematoxylin and eosin; SDH = succinic dehydrogenase.

In dog 1, EMG under general anesthesia revealed spontaneous abnormal activity in the interosseous palmar muscle (Figures 2 and 3 + positive sharp waves), interosseous plantar muscle (2 + polyspikes and increased insertional activity), and the cranial tibial and infrascapular muscles (1 + polyspikes and positive sharp waves). Other spontaneous activity was not recorded in the remainder of the muscles including limb muscles, paraspinal, masseter, and temporalis muscles. Motor nerve conduction velocities and CMAP were normal in the sciatic, ulnar, and radial nerves. Repetitive nerve stimulation did not reveal a decrement. These findings were compatible with a polymyopathy.

### Histopathology

Muscle biopsy samples were submitted from dog 1 to the Comparative Neuromuscular Laboratory from the triceps, infrascapular, paravertebral lumbar, cranial tibial, and semitendinosus muscles. Similar changes were present in all muscles and representative images from the paravertebral muscle are shown in Figure 3. Variability in myofiber size was present (Figure 3A) with atrophic type 2 fibers and a type 1 fiber predominance (Figure 3B). Numerous clear vacuoles were present in type 1 fibers that were evident with the ATPase reaction (Figure 3B). Prominent intramyofiber lipid droplets were present within type 1 fibers (Figure 3C). The combined mitochondrial specific reactions COX/SDH were performed and scattered COX negative muscle fibers were observed (Figure 3D). These findings were compatible with a metabolic myopathy.

### Genetic testing

**Figure 4:**
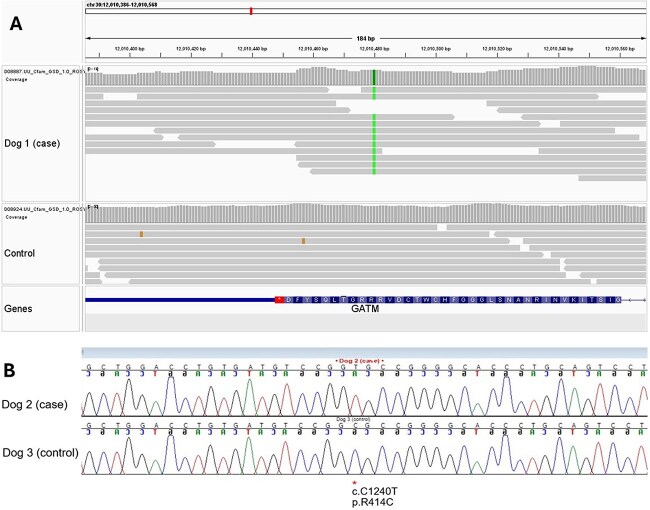
Identification of specific *GATM* variant. (A) IGV view of a homozygous G > A nucleotide substitution in dog 1 resulting in a *GATM* p.R414C (NP_001274013.1) missense variant; this variant is absent in a control dog. (B). Sanger sequencing chromatogram of dog 2 (case) and dog 3 (unaffected sibling); dog 2 was confirmed to be homozygous for the *GATM* p.R414C variant, while dog 3 was clear. Abbreviations: GATM = glycine amidinotransferase; IGV = integrative genomics viewer.

Variants obtained from WGS of dog 1 were compared to those of the internal WGS database to identify variants absent in the reference population. Variants were evaluated and prioritized based on impact (high or moderate) and gene function. A list of these unique variants is provided in Table S1. Dog 1 was found to have a unique homozygous missense variant in *GATM* (CanFam 4 chr30:12,010,480G > A; NP_001274013.1 p.R414C). Variant calling was confirmed by visual inspection using the integrative genomics viewer^15^ (Figure 4A). Follow-up genotyping with Sanger sequencing of the affected sibling (dog 2) confirmed homozygosity for the R414C variant; the unaffected sibling was clear (Figure 4B). The variant is predicted to be deleterious using in silico programs MutPred2 and PolyPhen2.^16^^,^^17^ Glycine amidinotransferase (*GATM*) variants have been associated with human creatine deficiency syndromes.^18^  *GATM* encodes arginine:glycine amidinotransferase (AGAT), a mitochondrial enzyme, and could explain the excessive intramyofiber lipid droplets and COX negative fibers observed with histochemical reactions in the muscle biopsies. These findings suggested an autosomal recessive mode of transmission with both parents suspected to be heterozygous for the *GATM* p.R414C allele.

### Treatment/outcome

Before receiving results of muscle biopsies and genetic testing, dog 1 was treated with L-carnitine (40 mg/kg BID), co-enzyme Q10 (4 mg/kg SID), and vitamin B (B3: 1 mg/kg/day; B5: 0.75 mg/kg/day; B6: 0.4 mg/kg/day; B1: 0.4 mg/kg/day; B2: 0.3 mg/kg/day; B9: 8 μg/kg/day; B7: 6 μg/kg/day; and B12: 2 μg/kg/day). A mild improvement in exercise tolerance was reported by the owners 1 month after starting supplementation. After withdrawal of L-carnitine, relapse of the exercise intolerance was noted by the owner to the level before supplementation. Treatment with L-carnitine was reinstituted with no further improvement noted and the dog remained at the same level of weakness as before initiating L-carnitine, co-enzyme Q10, and vitamin B supplementation.

After receipt of the muscle biopsy report, identification of the *GATM* variant in dog 1 and confirmation of the genotypes in dogs 2 and 3, a treatment trial with creatine was performed as described in a similar CDD myopathy in people.^19^ Dog 1 received a dose of 800 mg/kg/day of creatine given once daily by mouth that was slowly tapered after a week by a reduction of 25% of the dose every week to reach 125 mg/kg/day given once daily. L-carnitine and co-enzyme Q10 were administered at the same dose as earlier and vitamin B supplementations were withdrawn. Dog 2 received a dose of 1500 mg/kg/day of creatine given once daily and the dose decreased to 225 mg/kg/day given once daily after 2 months. Dog 2 also received L-carnitine supplementation (75 mg/kg twice a day), started at the same time as the creatine.

After addition of creatine to co-enzyme Q10 and L-carnitine supplementation, a marked improvement in the gait was reported in dog 1. Exercise intolerance resolved apparent as the ability to walk without weakness for more than 20 minutes. The dog was no longer reluctant to walk up the stairs. The bunny hopping gait remained while running but with a subjective improvement in the pace reported by the owners. Recheck examination at 4 months revealed a normal neurological examination except for a persistent bunny hopping gait while running. The dog started to jog with the owners without collapsing which it did before supplementation. Video S2 compares the gait of dog 1 from before and after supplementation.

Episodes of regurgitation resolved completely in both dogs within a month of supplementation. The postprandial hiccup sound reported in dog 1 was also resolved. Despite clinical resolution of the regurgitation, thoracic radiographs at 4 months after treatment revealed a persistent megaesophagus in dog 1.

Both dogs showed an improvement in weight. Dog 2, which was cachectic with marked muscle atrophy before treatment, gained 2 kg while dog 1 gained 1.4 kg over the 4-month period. Adverse effects of treatment were not reported in dog 2. Dog 1 had occasional vomiting episodes during the first week of treatment that resolved after dosages of creatine were reduced.

## Discussion

Here we describe cases of a CDD in dogs with a missense variant in *GATM* resulting in arginine:glycine amidinotransferase (AGAT) deficiency. Creatine deficiency disorder is a rare clinical syndrome in people that results from errors in creatine metabolism: from improper synthesis related to variants in the glycine amidinotransferase (*GATM)* gene, guanidinoacetate methyltransferase (*GAMT*) gene or from a creatine transporter (CRT) variant.^20^ The AGAT enzyme is located in the mitochondrial intermembrane space and has high levels in the brain and muscle. Hallmarks of CDD are signs of encephalopathy that manifests mainly with cognitive dysfunction with seizures being less common. Movement disorders occur in GAMT and CRT deficiency, while myopathy has only been reported in AGAT deficiency.^18–23^ In people, diagnosis is made by a combination of exclusion of other encephalopathies and myopathies, familial history and testing for a specific mutation. Lastly, supplementation with creatine results in a very favorable response with improvement of the encephalopathy reported in all 3 CDDs and improvement of the myopathy in the AGAT deficiency.

Creatine is an essential metabolite for cell metabolism. It serves as a substrate for the enzyme CK to phosphorylate creatine leading to the storage of a phosphoryl group (PO_3_^2-^) from adenosine triphosphate (ATP). This allows the phosphocreatine molecule created to rapidly restore ATP from adenosine diphosphate by the reverse enzymatic reaction and quickly restore the ATP pool when energy is needed. In people, creatine is synthesized in the liver, kidney, pancreas, and to a lesser extent in the brain and testis. Two main enzymatic reactions occur. L-arginine and glycine are converted into guanidinoacetate by arginine:glycine amidinotransferase (AGAT). Guanidinoacetate is then converted into creatine by the guanidinoacetate N-methyltransferase (GAMT) enzyme. Creatine is then released into the blood stream and is located primarily in skeletal muscles but also in other high energy demanding organs such as the brain. Intracellular transport of creatine is made through a carrier protein called sodium and chloride dependent CRT.^24^

To our knowledge, the three-dimensional structure of the AGAT enzyme is not known in the dog. However, the AGAT R414 residue, for which a variant of the encoding gene *GATM* was found on dogs 1 and 2, and which corresponds to an arginine in position 414 in the AGAT enzyme in human, is universally conserved across the hg38 human multiple alignments of 100 vertebrate species available on the UCSC Genome Browser^25^^,^^26^ with a phyloP score of 8.80 (score range [−20; 30]).^27^^,^^28^ In addition, within the Broad Institute’s gnomAD browser there are reports of 4 AGAT R414 residue encoding alleles (all heterozygous) out of 1 613 684 genomes of human.^29^ Finally, gnomAD also reports alleles of the immediately preceding residue AGAT R413 (R413W, R413Q) associated with AGAT deficiency in a human patient compound heterozygous for these variants.^30^ The universally conserved AGAT R414 (positive phyloP score) and the report of AGAT deficiency syndrome in human associated with the preceding residue AGAT R413 support the hypothesis that the AGAT R414 residue is playing an important role for the AGAT function in the dog as suspected in dogs 1 and 2 reported in this article.

The 2 dogs in this report had a *GATM* variant with a myopathy that manifested by marked exercise intolerance. Histopathology of muscle biopsies supported a metabolic myopathy with lipid storage and sporadic COX deficient muscle fibers. A CDD was identified based on genetic testing. The familial history of similar clinical signs since birth, and response to creatine supplementation, supported CDD. In people, measurement of guanidinoacetate and creatine in urine, cerebrospinal fluid or plasma can help differentiate AGAT deficiency from GAMT deficiency.^19^^,^^22^ Creatine concentration is low in both AGAT deficiency and GAMT deficiency and guanidinoacetate concentration is high in GAMT and low in AGAT deficiency. In our cases, guanidinoacetate and creatine levels could not be measured. Based on the excellent response to therapy and identification of the *GATM* variant, AGAT deficiency is likely causative.

Treatment with L-carnitine and creatine was initiated in both affected dogs with a marked improvement in exercise intolerance in dog 1 and resolution of regurgitation in both dogs. In dog 1, L-carnitine treatment was initiated before creatine and a mild improvement in exercise intolerance was noted with a deterioration after withdrawal. However, restarting the medication did not result in improvement, as reported by the owners. This response to L-carnitine supplementation suggests either a placebo effect or a short-term response with an acquired tolerance and therapeutic escape. L-carnitine functions to transport fatty acids into the mitochondria, indirectly increasing the rate of ß-oxidation.^31^ Despite the mild response noted with L-carnitine, a better improvement was reported by the owner of dog 1 on creatine supplementation. Both dogs continue to do well at the time of writing (4 months after diagnosis) while maintaining supplementation of both creatine and L-carnitine. These responses to treatment support a more important role of creatine as described in human CDD.

Several similarities with the human AGAT deficiency were observed in the affected dogs: signs of myopathy, a variant in the AGAT protein coding region (*GATM* gene), an autosomal recessive mode of transmission and a good response to creatine supplementation.^18^^,^^20^^,^^21^ Despite those similarities some differences were observed in the 2 dogs compared to their human counterparts.

Neither of the affected dogs presented with cognitive developmental delay, behavioral disorders, or seizures that are, respectively, reported in humans in 80%, 25%, and 10% of the cases.^20^ Both dogs were reported to have apparently normal cognition and training throughout their development and no behavioral disorder could be identified. However, cognitive dysfunction in dogs remains subjective and might only be revealed at later stages of disease. Brain magnetic resonance imaging (MRI) and electroencephalography (EEG) recordings were not performed to rule out a brain lesion or epileptic syndrome. In another human creatine deficiency disorder, GAMT deficiency, some patients are reported on MRI imaging with T2 hyperintense lesions compared to the white matter within brain basal ganglia, but could not be evaluated in our cases.^19^ A subclinical encephalopathy is therefore not excluded in the AGAT deficiency in dogs but might be clinically irrelevant in dogs compared to people.

Polymyopathy is the second most common clinical sign of AGAT deficiency in humans after cognitive developmental delay, occurring in 50% of the cases.^20^^,^^22^ In the 2 cases presented here, signs of muscle disease predominated. It is therefore possible that in dogs with AGAT deficiency, polymyopathy might be the primary clinical presentation. Megaesophagus was present in both affected dogs. Megaesophagus is associated with generalized myopathic syndromes in dogs and could represent a consistent finding in AGAT deficiency.^32^ In dogs, the esophagus is composed entirely of skeletal muscle and lacks smooth muscle whereas the human esophagus is composed of a combination of smooth and skeletal muscles.^33^ This anatomical difference might explain why esophageal dysfunction is observed in dogs but not in humans with AGAT deficiency.

The main limitation of our report is the small number of cases described. Rarity of this disease makes a complete clinical picture of AGAT deficiency in dogs challenging. While the response to creatine supplementation in humans is usually good, the response to treatment in dogs requires further documentation. A second limitation results from possible bias in the choice of diagnostic tests. Both dogs were presented for a clinical neurological examination and therefore testing was oriented for diagnostic purposes rather than documenting the disease. It is likely that some diagnostic tests such as brain MRI or EEG could have led to a more complete characterization of the disease but were not required for diagnosis and treatment planning. Finally, both affected dogs were monitored for a relatively short period of time (4 months). While a good long-term outcome is described in human AGAT deficiency, longer follow-up is required to know if a similar good outcome also occurs in dogs.

### Conclusion

Our study presents the characterization of a CDD associated with a novel *GATM* variant resulting in predominantly signs of muscle disease including exercise intolerance and esophageal dysfunction. Creatine and L-carnitine supplementation resulted in marked improvement in exercise intolerance and esophageal dysfunction. In addition, identification of this *GATM* variant expands the spectrum of causes of lipid storage in muscle and megaesophagus, thus adding an additional differential for these clinical presentations.

## Supplementary Material

aalaf055_Supplemental_video_1

aalaf055_Supplemental_video_2

aalaf055_Supplementary_Table_1_Table_containing_all_predicted_protein_changing_variants

## Off-label antimicrobial declaration

The authors declare no off-label use of antimicrobials.

## Institutional animal care and use committee or other approval declaration

The study was performed on client owned dogs with written and oral consent obtained for diagnostic procedures.

## Abbreviations

AGAT: arginine:glycine amidinotransferase protein
ATP: adenosine triphosphate
CDD: creatine deficiency disorder
CK: creatine kinase
CMAP: compound muscle action potential
COX: cytochrome c oxidase
CRT: creatine transporter
CT: computed tomography
EMG: electromyography
EEG: electroencephalography
GAMT: guanidinoacetate methyltransferase
GATM: glycine amidinotransferase gene
HU: Hounsfield unit
MRI: magnetic resonance imaging
SDH: succinate dehydrogenase
WGS: whole genome sequencing
