# Correction to: Myopathy due to a creatine deficiency disorder in a family of mixed breed dogs with a glycine amidinotransferase gene mutation

**DOI:** 10.1093/jvimsj/aalag102

**Published:** 2026-05-06

This is a correction to: Hugo Leonardi, Katie M Minor, Julien Fritz, Steven G Friedenberg, Jonah N Cullen, Ling T Guo, G Diane Shelton, Myopathy due to a creatine deficiency disorder in a family of mixed breed dogs with a glycine amidinotransferase gene mutation, *Journal of Veterinary Internal Medicine*, Volume 40, Issue 1, January-February 2026, aalaf055, https://doi.org/10.1093/jvimsj/aalaf055

In the originally published version of the paper, in section **Author Contributions** the name for Dr Shelton has been corrected to read: “G. Diane Shelton” instead of: “G. Diane Diane Shelton.”

The article was also missing materials from section **Supplementary data**: the Supplemental Video 2 and a Supplemental Table 1 are now supplied.

The publisher regrets these errors.

